# Employment interventions to assist people who experience borderline personality disorder: A scoping review

**DOI:** 10.1177/00207640231189424

**Published:** 2023-07-27

**Authors:** Jocelyn Kernot, Amy Baker, Candice Oster, Melissa Petrakis, Suzanne Dawson

**Affiliations:** 1 Allied Health & Human Performance Academic Unit, University of South Australia, Adelaide, Australia; 2 Caring Futures Institute, Flinders University, Bedford Park, SA, Australia; 3 Department of Social Work, Monash University, Melbourne, VIC, Australia; 4 Southern Adelaide Local Health Network, Adelaide, SA, Australia

**Keywords:** Borderline personality disorder, Individual Placement and Support, vocational support, employment, intervention, scoping review

## Abstract

**Background:**

Employment is an important social determinant of health and is associated with positive health outcomes. However, individuals who have been diagnosed with borderline personality disorder (BPD) are significantly underrepresented in the workforce. Whilst there is an array of evidence based therapeutic interventions, there remains a gap in knowledge regarding the most effective ways to enhance employment outcomes for people with a diagnosis of BPD.

**Aim:**

To explore employment interventions for people with BPD, map the available evidence and identify key concepts and knowledge gaps.

**Methods:**

A scoping review was conducted to identify and map the relevant literature. Findings were summarised using a narrative approach. Consultation was provided by a reference group including peer support workers with lived experience of BPD and mental health clinicians.

**Results:**

Seven articles met the inclusion criteria, including non-randomised and case study/series designs and a randomised controlled trial protocol, with participant numbers generally low. All programmes combined a psychotherapeutic component with work related goals; however, there were notable differences in relation to the conceptual/theoretical approach of the psychotherapeutic component and delivery of the work-related components. Barriers and enablers to programme participation and success are explored.

**Conclusions:**

This review provides important insights into the characteristics of vocational rehabilitation interventions for people diagnosed with BPD. The findings will inform the co-production of approaches to support people with BPD to engage in employment.

## Background

The clinical diagnosis of Borderline Personality Disorder (BPD) has been characterised by a person experiencing an extreme sensitivity to perceived interpersonal slights, an unstable sense of self, intense and volatile emotions and impulsive behaviours that are often self-destructive (American Psychiatric Association, 2022; Gunderson, Masland, & Choi-Kain, 2018). The diagnosis of BPD is controversial due to the stigma and therapeutic nihilism often associated with the diagnosis (Campbell et al., 2020). Consequently, in the UK, researchers and some mental health services are using the term ‘complex emotional needs’ to refer to individuals who may have received a diagnosis of BPD (Trevillion et al., 2022). In this scoping review we have used the more common term BPD to align with the relevant literature.

Approximately 15% to 28% of people receiving care in psychiatric outpatient clinics or hospitals and 1.7% of the general population have a diagnosis of BPD (Gunderson, Masland, & Choi-Kain, 2018). Various longitudinal studies examining outcomes for individuals with BPD report individuals continue to experience significant functional impairment. Studies highlight that whilst evidence-based psychotherapies may assist individuals in their recovery, current interventions are not leading to sustained functional outcomes (Choi-Kain et al., 2016; Soloff, 2021; Zanarini et al., 2010).

Employment is an important social determinant of health which is critical to improving individuals’ health outcomes (Hergenrather et al., 2015). Individuals with BPD report wanting better relationships and employment (Ng et al., 2019). Participation in work has been associated with positive outcomes for people with BPD, such as improved physical health (Cruitt et al., 2018), self-esteem and a sense of meaningful contribution (Grenyer et al., 2022). Thus, interventions to support participation in work have the potential to support people’s long-term functional recovery (Gunderson, Herpertz, Skodol, et al., 2018; Winsper, 2021). However, a review of studies exploring various work variables for people with BPD found that few studies addressed employment outcomes for people with BPD (Sansone & Sansone, 2012).

Most research exploring employment for people with BPD focusses on the extent to which occupational or vocational functioning has been impacted (Cruitt & Oltmanns, 2019). An integrative review exploring the impact of common symptoms related to BPD on occupational capacity, participation and sustainability reported that BPD ‘symptomatology and behaviours’ resulted in significant barriers to sustained and meaningful employment (Larcombe & Müller, 2022). Factors which may negatively impact on employment include self-injurious and self-sabotaging behaviour, poor affective stability and identity problems related to a BPD diagnosis (Thompson et al., 2012). Juurlink et al. (2019) reported that stigma surrounding a diagnosis of BPD affected employment, with participants emphasising that BPD should be relabelled to incorporate more positive terminology. Vocationally targeted interventions were recommended alongside standard treatment for young people with BPD, with such interventions to be introduced as early as possible (Juurlink et al., 2022). In a recent qualitative study exploring chronic feelings of emptiness experienced by people with BPD, the authors suggested that strengthening identity, sense of purpose and vocational functioning may reduce the intensity of feelings of emptiness, with several participants identifying employment as ‘a distraction from the feeling of emptiness’ (Miller et al., 2021, p. 5).

Whilst there are a range of evidence-based psychotherapy interventions for people with BPD, less is known about successful employment supports. The Individual Placement and Support (IPS) programme is the most developed and validated programme to support individuals with severe mental illness to gain and sustain employment (Bond et al., 2020). IPS adheres to eight practice principles: (1) focus on competitive employment, (2) zero exclusion, (3) focus on client work preferences, (4) rapid job search, (5) targeted job development, (6) integration of employment and mental health services, (7) benefits counselling and (8) ongoing support (Bond et al., 2020). Although IPS has been found to be effective with consumers with a range of mental health diagnoses, and there is research demonstrating improved results with augmented programmes (e.g. cognitive strategies) (McDowell et al., 2021), there is limited information regarding the effectiveness of the programme for people with BPD. Currently, there is no known review of the literature presenting the types of employment interventions for people with BPD, including the characteristics, employment outcomes, barriers and enablers reported for such interventions. The aim of this scoping review is to explore what employment interventions have been specifically implemented with people with BPD.

## Methods

This review was undertaken to inform a larger study looking at the co-design and co-production of strategies to support people with BPD to engage in an evidence-based employment intervention (Individual Placement and Support) and sustain employment.

## Incorporating co-design

The review was conducted in consultation with a reference group which included mental health clinicians (*n* = 4, with *n* = 3 involved in employment interventions) and peer support workers with lived experience of BPD (*n* = 2). Consultation was undertaken via two Zoom meetings during the conceptualisation phase to assist with formulating the research questions and to review/advise on the search terms, and then via two Zoom meetings in relation to categories developed from extracted data and to inform the discussion of implications to practice and future research.

## Research design

A scoping review was selected to identify the breadth of literature on this topic, map the available evidence and identify key concepts and knowledge gaps (Peters et al., 2020). The six-stage framework developed by Arksey and O’Malley (2005), including extensions described in the JBI Manual for Evidence Synthesis (Peters et al., 2020), were used to guide the review. The six stages were: (1) identify the research question, (2) identify relevant studies, (3) study selection, (4) charting the data, (5) collating, summarising and reporting results and (6) consultation (Peters et al., 2020).

## Identifying the research question

The primary research question was broad in nature – ‘What employment interventions for people with BPD have been reported in the literature?’ The following sub questions were formulated to assist with guiding data extraction – ‘What are the characteristics of these interventions?’; ‘What outcomes have been reported?’; ‘What were the barriers and enablers reported?’ and ‘How do the barriers and enablers map against the IPS practice principles?’

## Identify relevant studies

Peer reviewed literature was identified through a search of six electronic data bases (Medline, PsycINFO, Emcare, Cochrane Library, CINAHL, ProQuest and Scopus; search date 14/4/22). The search strategy was developed in consultation with an academic librarian and conducted in Medline (see Supplemental Material) and then adapted for other databases. Search terms included ‘borderline personality disorder’, or ‘emotionally unstable personality disorder’, or ‘impulsive personality type’, or ‘borderline type’, or ‘borderline disorder’ or ‘Cluster B’ and ‘vocational rehabilitation’, or ‘vocation*’, or ‘employment’, or ‘individual placement and support’. A search of grey literature was also conducted via ProQuest, Trove and Google. All searches were limited to English language with no limits based on year of publication.

## Study selection

Search results were exported into Endnote (Version 20) where duplicates were removed. Results were then exported to Covidence (2022 version), where further removal of duplicates occurred for title/abstract and full text screening. Screening of title and abstracts and subsequent full texts were undertaken by two independent reviewers (JK and AB) with conflicts resolved by a third reviewer (CO). Study selection was made according to the following inclusion and exclusion criteria.

## Inclusion criteria

Publications were included if they were written in English and outlined an employment intervention for people with BPD. If studies included people with several diagnoses, at least 50% of the sample had to have a diagnosis of BPD. There were no exclusions based on study design or age of participants.

## Exclusion criteria

Conference abstracts were excluded as these were unlikely to provide enough detail about the employment intervention. Interventions specifically focussed on further education or volunteer employment were also excluded, with the focus of this review being on interventions designed to assist people to gain and maintain paid employment.

## Charting the data

Data was charted/extracted by two independent reviewers (JK and CO) using a customised data extraction template developed in Covidence; with consensus through review and discussion (JK, CO). Data extraction categories included year of publication, title, authors, country (research/intervention was conducted), aim of study, study design, participant characteristics (number, age, gender, diagnoses), recruitment methods, intervention characteristics (name, duration, frequency, activities, staffing, funding), barriers and facilitators to intervention participation, outcome measures and outcomes.

## Collating, reporting and summarising the findings

This review used a narrative approach to collate and summarise findings according to pre-existing categories related to the research questions. Specific consideration was given to the similarities and differences in intervention characteristics and barriers/facilitators as this informed a larger study looking at the practical application of strategies to assist people with BPD to engage in an employment intervention.

## Results

**Figure 1.**
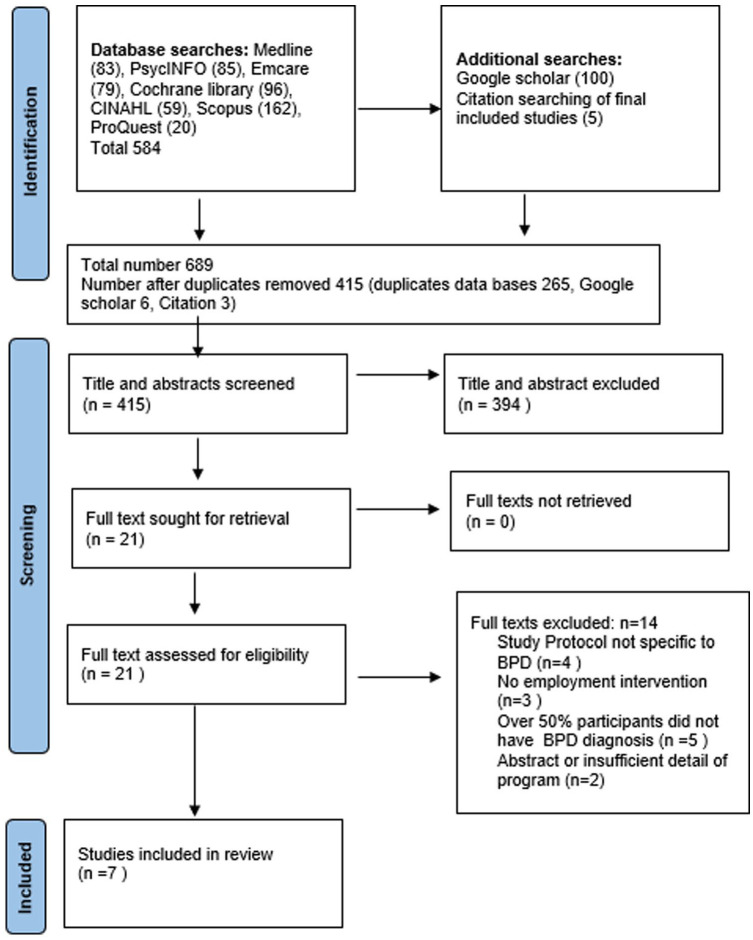
PRISMA flow diagram of search strategy and included studies.

The search identified 584 publications, seven of which met the inclusion criteria (Figure 1).

Table 1 presents a summary of articles included in the scoping review. All seven of the included articles were peer reviewed and published in psychiatric/mental health or vocational rehabilitation journals between 2010 and 2020, except for a study protocol published in the Cochrane Central Register of Controlled Trials. Five of the included studies were conducted in the United States of America, with one each in Canada and Australia. Study designs included case studies/case series (*n* = 3) (Dahl et al., 2017; Elliott & Konet, 2014; Gaztambide, 2019), non-randomised experimental designs (*n* = 2) (Comtois et al., 2010; Koons et al., 2006), randomised controlled trial protocol (*n* = 1) (Chanen et al., 2020) and an intervention report (*n* = 1) (Elliott & Weissenborn, 2010). Participant numbers ranged from 3 to 85, with the mean age of participants ranging from 30 to 40.7 years, with women being the predominant participant group.

**Table 1.** Study design, interventions and outcome measures in relevant studies. Summary of included articles.

Author, year, country	Study design	Participant	Intervention name, duration/frequency	Intervention activities	Outcome measures	Intervention outcomes
Chanen et al. (2020) Australia	Protocol for a randomised control trial	Young people 15–25 years with BPD	IPS (over 39 weeks) integrated with the HYPE programme (6–7 months, with up to 4 follow appointments over subsequent 6 months)	IPS. HYPE – psychiatric care, case management and time-limited psychotherapy (Cognitive Analytic Therapy)	Number of days in employment or education from baseline, cost effectiveness, BPD severity- BPDSI-IV	N/A
Comtois et al. (2010) USA	Non-randomised experimental study. Feasibility trial	30 participants, mean age 37 years (*SD* 11), 24 were women (80%), 29 had BPD (97%)	SDBT (1 year) and DBT-ACES (1 year)	SDPT as per treatment manuals. DBT-ACES included: individual therapy, graduated work requirement of 10 hr or 20 hr of work/week of paid work or enrolment in vocational education, group sessions	Abbreviated version of Quality of Life (QoL) Interview. Employment/education outcomes. Treatment History Interview	↑odds of being employed ⩾20 hr per week (*OR* = 4.93, *p* = .01) or in education (*OR* = 3.34, *p* = .05); ↑in subjective QoL (*B* = 0.49, *p* = .3); ↓in inpatient admissions (*RR* = .07, *p* < .05). 1 year after DBT-ACES only 36% of participants were receiving public mental health services
Dahl et al. (2017) Canada	Qualitative multiple case study	9 participants (8 women) with BPD, mean age 30 (*SD* = 8) Each participant to have a goal related to work (i.e. gaining work or return to work)	Not reported	Specialised work reintegration programme (based on IPS) + DBT or Mentalisation-Based Treatment (MBT) with aim to develop emotional and relational capacities.	Qualitative interviews, sociodemographic questionnaire	Individual factors (e.g. reactions to pressure, work relationships and emotional regulation) and factors related to insurance and organisation/health systems (e.g. poor communication between stakeholders, work accommodations and work-place support) impacted on work participation
Elliott & Konet (2014) USA	Case series. Pilot project	54 participants with BPD, most were female (80%). Mean age 36 years (range 19–62)	TCP Programme run for 16-week, 2 days/week for 5 hr/day	Group meetings with two co-leaders and 5–10 participant; Individual meetings with a vocational coach once per week; Internship at TCP (varied work roles); Free time on computers	Progress was evaluated in relation to: (1) those who gained employment or similar goals (2) those who significantly improved their job preparedness (e.g. internship and completed TCP curriculum) (3) Those who made some/limited progress towards job preparedness	26 participants obtained full time employment or similar goals, 12 improved their job preparedness, 14 made limited progress towards job preparedness and two participants made no progress
Elliott & Weissenborn (2010) USA	Brief report	85 participants (age and gender not reported)	TCP Programme run for 16-week, 2 days/week for 5 hr/day	Group meetings with two co-leaders and 5–10 participants; Individual meetings with a vocational coach once per week; Internship at TCP (varied work roles); Free time on computers	Not Applicable	Not Applicable
Gaztambide (2019) USA	Case series	3 participants (1 female middle aged, 2 males one late middle aged and 1 young adult)	Transference-Focused Psychotherapy (TFP). Duration and frequency not reported	Focused on persons’ ability to negotiate problems in the social world. It included 2 components (a) contracting and planning around safety (b) person engaging in activity outside the session through paid or volunteer work to allow exploration of relational experiences	Not Applicable	Not Applicable
Koons et al. (2006) USA	Non-randomised experimental study	12 participants, mean age 40.7 year (*SD* = 7.95), 11 women and 1 man	Dialetical Behaviour Therapy for Vocational Rehabilitation. 6-month programme, delivered in a weekly 4-hr block with 2 hr of standard DBT and a 90 min group focussed on getting and keeping a job	Standard DBT included the distress tolerance, emotion regulation and interpersonal effectiveness skills modules and mindfulness skills modules. Teaching metaphors, role-plays and homework was provided on obtaining and sustaining employment. Participants kept two diary cards one for standard DBT and one employment related targets. DBT leaders met weekly for 1 hr for case consultation	Social Adjustment Scale (self-report version), Beck Depression Inventory, Beck Hopelessness Scale, State-trait Anger Inventory; assessed at completion of treatment and 6 month follow up. Hours of employment, weekly employment, work role satisfaction and employment income; measured at completion of treatment and 6 and 12 month follow up	Participants who completed the programme (*n* = 8) had significant improvement (*p* < .05) in depression, hopelessness and experience of anger and maintained this at 6 month follow up. At 6 month follow up significant improvement (*p* < .05) was noted in anger expression, control of anger expression, work role satisfaction and number of hours worked weekly

*Note*. HYPE = Helping Young People Early Programme; SDBT = Standard DBT; DBT-ACES = Dialectical Behaviour Therapy Accepting the Challenges of Exiting the System; TCP = The Connections Place.

## Characteristics of employment interventions

Interventions tended to occur over a time frame of 6 to 12 months and include weekly input, with The Connection to Place (TCP) programme having the shortest duration of 16 weeks with twice weekly input, 5 hr per day (Elliott & Konet, 2014; Elliott & Weissenborn, 2010). All interventions were multi-modal, with three including both individual and group sessions (Comtois et al., 2010; Elliott & Konet, 2014; Elliott & Weissenborn, 2010; Koons et al., 2006) and all including a work (with work related goals/experiences) and an emotional psycho-therapeutic component. Tools used to facilitate group or individual sessions included learning modules, having a primary coach, goal setting, individualised support plans, verbal contracts, role playing, homework and diary cards (to monitor progress). All programmes featured structures or frameworks that identified treatment expectations (time frames and intensity) with some outlining consequences if expectations were not met (e.g. discontinuation or a break from the programme). While clients were actively involved in their individual goal setting, no information was provided on clients’ input into programme content, design or scheduling. Active involvement from the therapist in supporting consumers’ goals, learning and problem solving was a feature of all programmes, with one programme (Comtois et al., 2010) including a group session on strategies to assist consumers with building relationships with health professionals. Providing opportunities for therapists to debrief or receive mentoring from others was also noted in three programmes (Chanen et al., 2020; Elliott & Konet, 2014; Elliott & Weissenborn, 2010; Koons et al., 2006).

Many of the programmes included extensive vocational support (Chanen et al., 2020; Dahl et al., 2017; Elliott & Konet, 2014; Elliott & Weissenborn, 2010). One programme featured the Individual Placement Support Programme (IPS) (Chanen et al., 2020), while another reported that their intervention was based on IPS (Dahl et al., 2017). Others shared similar features to IPS with vocational coaching (Elliott & Konet, 2014; Elliott & Weissenborn, 2010; Koons et al., 2006) to prepare participants for employment. This included discussion and practice around preparing a Curriculum Vitae (CV), writing a job application, preparation for /role playing interviews, guest speakers and job seeking support. Other programmes (Comtois et al., 2010; Gaztambide, 2019) did not have employment preparation or vocational coaching but instead required participants to have employment goals and to participate in graded paid or volunteer employment with the focus being on the psycho-therapeutic component.

The conceptual/theoretical approaches for the emotional psychotherapeutic component varied across programmes, featuring Dialectical Behavioural Therapy (DBT) (Comtois et al., 2010; Dahl et al., 2017; Koons et al., 2006) (*n* = 3), Mentalisation-Based Treatment (Dahl et al., 2017), Cognitive Analytical Therapy (HYPE programme; Chanen et al., 2020) and Transference-Focused Psychotherapy (Gaztambide, 2019). Commonalities included a focus on relationships, emotional responses/regulation, challenging negative thought processes and supported problem solving.

## Outcomes

Employment outcomes were a predominant feature of programme evaluation. Two studies included non-randomised experimental designs. Both programmes incorporated DBT and reported significant improvement in the numbers of hours worked/week at 6-month follow up (Koons et al., 2006), or odds of being employed at least 20 hr per week at the completion of the programme (Comtois et al., 2010). In a pilot study of The Connections Place programme, 48% of participants (26/54 clients) gained full time employment or a similar goal (i.e. full-time or part-time employment, full-time enrolment in school or full-time internship) and 22% (12/54) improved their job preparedness (Elliott, 2014). In relation to non-employment outcomes, Comtois et al. (2010) reported a significant improvement in quality of life and a decrease in inpatient admissions. In contrast, Koons et al. (2006) explored the impact of the programme on depressive symptoms (Beck Depression Inventory), hopelessness (Beck Hopelessness Scale) and anger (State-trait Anger Inventory) and reported a significant improvement in all three at programme completion and 6-month follow up.

## Barriers and facilitators

The following barriers were identified at the individual, programme structural and systems level that may impede the success of a vocational programme:

(1) Individual level: the person’s ability to manage emotions and social skills at work, work stresses and low self-esteem (Dahl et al., 2017), and substantial increase in distress as a return to work or an increase in hours at work got nearer (Comtois et al., 2010).
(2) Programme structure: variation in participants’ time in a programme and dropout rates – with programme activities requiring adaptions to accommodate this (Elliott & Konet, 2014; Elliott & Weissenborn, 2010); programme contracts not emphasising consumers investment in gaining employment (Gaztambide, 2019).
(3) System level: challenges in attending therapy while working, insufficient work opportunities, poor communication and coordination between all stakeholders and difficulties accessing supports such as financial assistance or work re-integration programmes (Dahl et al., 2017).

In contrast, at the programme structural level there were various programme enablers and strengths which supported consumers participation in vocational rehabilitation programmes, including:

(1) Programme design which specifically addressed the needs of consumers with BPD, particularly in group programmes where participants could offer support to each other (Elliott & Konet, 2014; Elliott & Weissenborn, 2010).
(2) Including an emotional/psychotherapeutic component to the programme to assist participants with managing and problem-solving work-related stressors (Chanen et al., 2020; Elliot & Konet, 2014; Elliott & Weissenborn, 2010).
(3) Good communication and coordination between key stakeholders and other therapies. (Dahl et al., 2017).
(4) Programme structures such as location in a building with other business rather than a mental health setting and programme scheduling (e.g. 2 days per week, internship at programme site) to assists with developing work routines (sleep, medication, time management, dress etc) (Elliott & Konet, 2014; Elliott & Weissenborn, 2010).

System level enablers included matching individuals’ suitability to the workplace and having appropriate accommodations and supports in place (e.g. early sick leave with clear structures for reintegration back to work) (Dahl et al., 2017).

These enablers and barriers were mapped against the principles of the IPS programme to identify the potential gaps and areas for IPS programme development (see Table 2).

**Table 2.** Mapping of IPS principles against enablers/barriers for engagement in vocational rehabilitation interventions identified in this review.

IPS Principles	Programme specifically addresses the needs of person with BPD including peer support	Inclusion of an emotional psychotherapeutic component	Consideration of suitability to work-place	Having appropriate accommodations and supports in place	Good communication & coordination between stakeholders	Programme in non-mental health setting	Programme scheduling to assist with developing work routines	Address increased feelings of distress as return to work or increased work hours get nearer	Adapt to individual needs
Competitive employment	–	–	–	–	–	–	–	–	–
Zero exclusion	–	–	–		–	–	–	–	–
Jobs related to preferences	Y	–	Y	Y	–	–	–	–	Y
Rapid job search	–	–	–	–	–	–	–	–	–
Employment specialist integrated with clinical team	–	–	–	–	Y	–	–	Y	–
Connection with employers based on work preferences	–	–	Y	–	Y	–	–	–	–
Ongoing individualised support for person and employer	Y	–	Y	Y	Y	–	–	Y	Y
Benefits counselling	–	–	–	Y		–	–	–	–

## Discussion

This scoping review identified seven articles that described vocational rehabilitation programmes (six programmes in total) for people diagnosed with borderline personality disorder. Participant numbers were generally low (ranging from 3 to 85) with study designs lacking control groups and randomisation. All programmes included an emotional psychotherapeutic component in conjunction with work-related goals. There were differences in relation to the conceptual/theoretical approach of the emotional psychotherapeutic component, with dialectical behaviour therapy being the most common (*n* = 3) (Dahl et al., 2017; Elliott & Konet, 2014; Gaztambide, 2019). In addition, there were differences in how the work-related components were delivered, with four offering extensive vocational support (two including/based on the IPS model) (Chanen et al., 2020; Dahl et al., 2017) and two requiring participants to have work goals but focussing on emotional/relationship experiences at work (Comtois et al., 2010; Gaztambide, 2019).

Barriers and enablers to programme participation related to individual factors (e.g. suitability to the workplace), programme structural elements (e.g. location, scheduling and framing of consumers goals/participation) and system level issues (e.g. consistency of coordination and communication between stakeholders). There has been significant investigation of barriers and facilitators to successful implementation of vocational rehabilitation programmes, with a focus on IPS (Boardman & Rinaldi, 2013; Bonfils et al., 2017; Moe et al., 2021), which would impact on programme participation. Some findings aligned across studies. For example, a key facilitator to successful IPS implementation is prioritising IPS participants’ work goals and hours (Bonfils et al., 2017), which was identified as a potential programme structural barrier for successful participant outcomes in our review. Whilst the various individual factors situate the problem with the person, programme and systems-level enablers could address the various challenges that individuals may face in finding and maintaining employment. Attitudinal (e.g. low staff expectations) and structural factors (e.g. employers willingness to employ someone with a mental illness) have been found to be the more significant barriers (Boardman & Rinaldi, 2013). The need to focus on structural and systemic issues impacting people with a diagnosis of BPD (having complex emotional needs), and specifically, broadening the scope of interventions to include social and occupational supports, has been identified in recent research (Foye et al., 2022; Trevillion et al., 2022).

Augmenting vocational rehabilitation programme goals with other interventions was a predominant feature of the programmes. The use of augmented interventions is supported in the literature with evidence suggesting that skills training (e.g. work group skills training and work-related social skills training) (McDowell et al., 2021) and cognitive and psychological interventions (Dewa et al., 2018; McDowell et al., 2021), in conjunction with employment support (i.e. IPS), may enhance job retention of people with severe or enduring mental illness (Dewa et al., 2018; McDowell et al., 2021). Additional augmentations to vocational rehabilitation that have been explored in the literature did not feature in the programmes described in this review. These include the use of technological tools such as apps to enhance employment programmes (McDowell et al., 2021; Nicholson et al., 2017), which could provide ‘follow-along’ support for consumers when they commence employment to monitor progress and to provide timely assistance – such as the WorkingWell mobile phone app designed by Nicholson et al. (2017). Similarly enhancing IPS through the integration of natural supports and supported education has also been advocated (Murphy et al., 2005). Natural supports are those which already exist in the work-place such as supervisors, co-workers, etc., who may be able to assist informally or formally with skill acquisition, feedback and social inclusion (Murphy et al., 2005).

As previously highlighted, the small sample sizes and lack of randomised and controlled study designs were a feature of the included studies. In addition, there was variation in the outcome measures used, although most included employment related outcomes including work obtainment, hours per week, income, work preparedness and satisfaction with work roles. A meta-analysis by Twamley et al. (2003) supports measurement of these constructs and also suggests including the duration of employment. The impact of employment on non-vocational outcomes is also highlighted in the literature (Bond et al., 2001; Twamley et al., 2003), with studies in this review measuring quality of life, severity of BPD, social adjustment, mood, anger and feelings of hopelessness. Self-esteem is another construct included in previous research (Bond et al., 2001; Torrey et al., 2000) but not featured in studies in this review.

Lockett et al., (2018) have developed a conceptual framework to enhance the performance of vocational rehabilitation programmes for people with mental illness. While specifically related to IPS, it highlights useful considerations for other vocational rehabilitation approaches (with many not addressed in the studies in this review). Eight programme dimensions (in addition to programme fidelity and vocational outcomes) impacting on programme performance are identified: (1) employment specialist expertise (skills, attitudes and knowledge e.g. BAKES scale), (2) programme intensity (length of time in programme, frequency of face to face contacts with employment specialist, number of contacts employment specialist has with mental health team and employers); (3) quality of programme delivery (i.e. quality of relationship between employment specialist and participant); (4) quality of mental health treatment (measuring the type and extent of mental health services and other social support); (5) programme feedback; (6) technical assistance; (7) removal of non-evidence based components; and (8) participant responsiveness (participant engagement and interest). These eight dimensions present important considerations for future programme developers and evaluators to facilitate enhanced/evidence-based programme implementation. Fidelity reviews are considered crucial to the quality and effectiveness of IPS (Shepherd et al., 2012), and as such, provide another avenue for evaluation of the programme.

The synthesis of findings from this review will be used to assist with the co-design of an IPS programme for people with BPD in South Australia. Augmented IPS programmes (e.g. inclusion of skills training, cognitive and psychological interventions) have been trialled and demonstrate the potential for additional gains in job outcomes for participating individuals (Dewa et al., 2018; McDowell et al., 2021). The mapping of the review findings against the IPS principles highlighted that further exploration of the specific needs of people with BPD is required and will be a feature of the co-design process with interviews with consumers and family members planned to explore their experiences and suggestions for supports that would be beneficial in gaining and maintaining employment. Inclusion of ongoing peer support from others with BPD was highlighted as a key facilitator with group interventions mentioned. This is not a current feature of the IPS programme and will be an important consideration for the co-design panel. Conducting the programme in a non-mental health setting was also raised as a facilitator, which conflicts with the IPS principle of integration with mental health services.

Based on the findings of this review, augmentation of the IPS programme would be recommended for consideration by the co-design panel to strengthen the exploration of programme attendees’ thoughts, feelings and affect in relation to employment. This may involve inclusion of an emotional psychotherapeutic component as was the featured in the studies in this review, with most including evidence-based treatment approaches (i.e. DBT, MBT, TFP) for BPD (Weinberg et al., 2011). These approaches were used in the vocational rehabilitation programmes to explore emotional responses/regulation and relationships, with many looking at application and problem solving in the work environment. Specific tools used to aid skill development could also be drawn upon in the design of a programme, including the use of learning modules, role play and diary cards.

Some programmes included therapy breaks if attendance or employment goals were not met, which is in conflict with IPS principles (i.e. not time limited). Many of these programmes were time intensive for the therapist and consumers (e.g. DBT-ACES individual therapy and weekly group sessions for a year) and consideration would need to be given to resources and balancing therapy with participants’ other commitments (including work). Evaluation will be crucial to inform programme refinement and to build on the research in this area. In addition to considerations mentioned earlier (i.e. Lockett et al. (2018) conceptual framework to enhance the performance of vocational rehabilitation programmes), Dewa et al. (2018) suggest that future researchers/programme developers of augmented IPS programmes should consider a process evaluation (i.e. to determine which processes are most effective); the timing of the augmentation (i.e. when during the employment journey augmentation would be most beneficial/effective); clear documentation of how augmentation impacts IPS fidelity; and maintaining individualised services (i.e. augmented programme maintains individualised choices). These will also inform co-design to ensure plans are in place for ongoing monitoring and evaluation of an augmented IPS programme. Additionally, factors contributing to successful IPS implementation will also be considered (Bonfils et al., 2017; Moe et al., 2021).

### Limitations

A key limitation is the low quality of the research identified in this scoping review in terms of both the methodologies used and the numbers of participants included in the studies. The inclusion of only English language articles is a further limitation.

## Conclusion

Workforce participation is an important social determinant of health and is associated with positive outcomes for people diagnosed with BPD. This scoping review provides important insights into the characteristics of vocational rehabilitation interventions for people diagnosed with BPD to inform future interventions and research. Vocational rehabilitation/supports in combination with an emotional psychotherapeutic component was a key element of all studies, highlighting the importance of a combined approach. Importantly, while individual level barriers were identified, this scoping review highlights the importance of addressing structural barriers to better support people with BPD to enter and maintain engagement with the workforce. There is a clear need for further (and high quality) research to explore how best to support people diagnosed with BPD to find and sustain work. This review will inform the co-production of strategies to address system level barriers and to support people with BPD to successfully engage in IPS.

## Supplemental Material

sj-docx-1-isp-10.1177_00207640231189424 – Supplemental material for Employment interventions to assist people who experience borderline personality disorder: A scoping review Click here for additional data file. Supplemental material, sj-docx-1-isp-10.1177_00207640231189424 for Employment interventions to assist people who experience borderline personality disorder: A scoping review by Jocelyn Kernot, Amy Baker, Candice Oster, Melissa Petrakis and Suzanne Dawson in International Journal of Social Psychiatry

## References

[bibr1-00207640231189424] American Psychiatric Association. (2022). Diagnostic and statistical manual of mental disorders, fifth edition, text revision (Vol. 5).

[bibr2-00207640231189424] Arksey H. O’Malley L. (2005). Scoping studies: Towards a methodological framework. International Journal of Social Research Methodology, 8(1), 19–32.

[bibr3-00207640231189424] Boardman J. Rinaldi M. (2013). Difficulties in implementing supported employment for people with severe mental health problems. British Journal of Psychiatry, 203(3), 247–249. 10.1192/bjp.bp.112.121962 24085736

[bibr4-00207640231189424] Bond G. R. Drake R. E. Becker D. R. (2020). An update on Individual Placement and Support. World Psychiatry, 19(3), 390–391. 10.1002/wps.20784 32931093 PMC7491619

[bibr5-00207640231189424] Bond G. R. Resnick S. G. Drake R. E. Xie H. McHugo G. J. Bebout R. R. (2001). Does competitive employment improve nonvocational outcomes for people with severe mental illness? Journal of Consulting and Clinical Psychology, 69(3), 489. 11495178 10.1037//0022-006x.69.3.489

[bibr6-00207640231189424] Bonfils I. S. Hansen H. Dalum H. S. Eplov L. F. (2017) Implementation of the individual placement and support approach – Facilitators and barriers. Scandinavian Journal of Disability Research, 19(4), 318–333. 10.1080/15017419.2016.1222306

[bibr7-00207640231189424] Campbell K. Clarke K. A. Massey D. Lakeman R. (2020). Borderline Personality Disorder: To diagnose or not to diagnose? That is the question. International Journal of Mental Health Nursing, 29(5), 972–981. 10.1111/inm.12737 32426937

[bibr8-00207640231189424] Chanen A. M. Nicol K. Betts J. K. Bond G. R. Mihalopoulos C. Jackson H. J. Thompson K. N. Jovev M. Yuen H. P. Chinnery G. (2020). INdividual Vocational and Educational Support Trial (INVEST) for young people with borderline personality disorder: Study protocol for a randomised controlled trial. Trials, 21, 1–12. 32591007 10.1186/s13063-020-04471-3 PMC7320570

[bibr9-00207640231189424] Choi-Kain L. W. Albert E. B. Gunderson J. G. (2016). Evidence-based treatments for borderline personality disorder: Implementation, integration, and stepped care. Harvard Review of Psychiatry, 24(5), 342–356. 27603742 10.1097/HRP.0000000000000113

[bibr10-00207640231189424] Comtois K. A. Kerbrat A. H. Atkins D. C. Harned M. S. Elwood L. (2010). Recovery from disability for individuals with borderline personality disorder: A feasibility trial of DBT-ACES. Psychiatric Services, 61(11), 1106–1111. 21041349 10.1176/ps.2010.61.11.1106

[bibr11-00207640231189424] Cruitt P. J. Boudreaux M. J. Jackson J. J. Oltmanns T. F. (2018). Borderline personality pathology and physical health: The role of employment. Personality Disorders: Theory, Research, and Treatment, 9(1), 73. 10.1037/per0000211 PMC5311027 27657166

[bibr12-00207640231189424] Cruitt P. J. Oltmanns T. F. (2019). Unemployment and the relationship between borderline personality pathology and health. Journal of Research in Personality, 82, 103863. 32863466 10.1016/j.jrp.2019.103863 PMC7448725

[bibr13-00207640231189424] Dahl K. Larivière N. Corbière M. (2017). Work participation of individuals with borderline personality disorder: A multiple case study. Journal of Vocational Rehabilitation, 46(3), 377–388.

[bibr14-00207640231189424] Dewa C. S. Loong D. Trojanowski L. Bonato S. (2018). The effectiveness of augmented versus standard individual placement and support programs in terms of employment: A systematic literature review. Journal of Mental Health, 27(2), 174–183. 28488948 10.1080/09638237.2017.1322180

[bibr15-00207640231189424] Elliott B. Konet R. J. (2014). The connections place: A job preparedness program for individuals with borderline personality disorder. Community Mental Health Journal, 50(1), 41–45. 23408295 10.1007/s10597-013-9601-y

[bibr16-00207640231189424] Elliott B. Weissenborn O. (2010). Employment for persons with borderline personality disorder. Psychiatric Services, 61(4), 417–417. 20360285 10.1176/ps.2010.61.4.417

[bibr17-00207640231189424] Foye U. Stuart R. Trevillion K. Oram S. Allen D. Broeckelmann E. Jeffreys S. Jeynes T. Crawford M. J. Moran P. McNicholas S. Billings J. Dale O. Simpson A. Johnson S. (2022). Clinician views on best practice community care for people with complex emotional needs and how it can be achieved: A qualitative study. BMC Psychiatry, 22(1), 72. 10.1186/s12888-022-03711-x PMC8796601 35090418

[bibr18-00207640231189424] Gaztambide D. J. (2019). Lines of advance in treating people of color with borderline personality disorder: Alloying the “gold” of vocational rehabilitation with the “copper” of psychodynamic psychotherapy. Psychoanalytic Social Work, 26(1), 50–68.

[bibr19-00207640231189424] Grenyer B. F. Townsend M. L. Lewis K. Day N. (2022). To love and work: A longitudinal study of everyday life factors in recovery from borderline personality disorder. Personality and Mental Health, 16(2), 138–154. 35538561 10.1002/pmh.1547 PMC9287094

[bibr20-00207640231189424] Gunderson J. G. Herpertz S. C. Skodol A. E. Torgersen S. Zanarini M. C. (2018). Borderline personality disorder. Nature Reviews Disease Primers, 4(1), 1–20. 10.1038/nrdp.2018.29 29795363

[bibr21-00207640231189424] Gunderson J. G. Masland S. Choi-Kain L. (2018). Good psychiatric management: A review. Current Opinion in Psychology, 21, 127–131. 29547739 10.1016/j.copsyc.2017.12.006

[bibr22-00207640231189424] Gunderson J. G. Stout R. L. McGlashan T. H. Shea M. T. Morey L. C. Grilo C. M. Zanarini M. C. Yen S. Markowitz J. C. Sanislow C. (2011). Ten-year course of borderline personality disorder: Psychopathology and function from the Collaborative Longitudinal Personality Disorders study. Archives of General Psychiatry, 68(8), 827–837. 21464343 10.1001/archgenpsychiatry.2011.37 PMC3158489

[bibr23-00207640231189424] Hergenrather K. C. Zeglin R. J. McGuire-Kuletz M. Rhodes S. D. (2015). Employment as a social determinant of health: A review of longitudinal studies exploring the relationship between employment status and mental health. Rehabilitation Research, Policy, and Education, 29(3), 261–290.

[bibr24-00207640231189424] Juurlink T. T. Betts J. K. Nicol K. Lamers F. Beekman A. T. Cotton S. M. Chanen A. M. (2022). Characteristics and predictors of educational and occupational disengagement among outpatient youth with borderline personality disorder. Journal of Personality Disorders, 36(1), 116–128. 34427492 10.1521/pedi_2021_35_534

[bibr25-00207640231189424] Juurlink T. T. Vukadin M. Stringer B. Westerman M. J. Lamers F. Anema J. R. Beekman A. T. Van Marle H. J. (2019). Barriers and facilitators to employment in borderline personality disorder: A qualitative study among patients, mental health practitioners and insurance physicians. PLoS ONE, 14(7), e0220233. 10.1371/journal.pone.0220233 PMC6650068 31335909

[bibr26-00207640231189424] Koons C. R. Chapman A. L. Betts B. B. Morse N. Robins C. J. (2006). Dialectical behavior therapy adapted for the vocational rehabilitation of significantly disabled mentally ill adults. Cognitive and Behavioral Practice, 13(2), 146–156.

[bibr27-00207640231189424] Larcombe E. Müller A. (2022). The relationship between borderline personality disorder and occupational participation: An integrative review. International Journal of Mental Health Nursing, 31(5), 1141–1150. 35536729 10.1111/inm.13014

[bibr28-00207640231189424] Lockett H. Waghorn G. Kydd R. (2018). A framework for improving the effectiveness of evidence-based practices in vocational rehabilitation. Journal of Vocational Rehabilitation, 49(1), 15–31.

[bibr29-00207640231189424] McDowell C. Ennals P. Fossey E. (2021). Vocational service models and approaches to improve job tenure of people with severe and enduring mental illness: A narrative review. Frontiers in Psychiatry, 12, 668716. 34305676 10.3389/fpsyt.2021.668716 PMC8298859

[bibr30-00207640231189424] Miller C. E. Townsend M. L. Grenyer B. F. (2021). Understanding chronic feelings of emptiness in borderline personality disorder: A qualitative study. Borderline Personality Disorder and Emotion Dysregulation, 8, 1–9. 34365966 10.1186/s40479-021-00164-8 PMC8351135

[bibr31-00207640231189424] Moe C. Brinchmann B. Rasmussen L. Brandseth O. L. McDaid D. Killackey E. Rinaldi M. Borg M. Mykletun A. (2021). Implementing individual placement and support (IPS): The experiences of employment specialists in the early implementation phase of IPS in Northern Norway. The IPSNOR study. BMC Psychiatry, 21(1), 632. 10.1186/s12888-021-03644-x 34930203 PMC8690340

[bibr32-00207640231189424] Murphy A. A. Mullen M. G. Spagnolo A. B. (2005). Enhancing individual placement and support: Promoting job tenure by integrating natural supports and supported education. American Journal of Psychiatric Rehabilitation, 8(1), 37–61.

[bibr33-00207640231189424] Ng F. Y. Carter P. E. Bourke M. E. Grenyer B. F. (2019). What do individuals with borderline personality disorder want from treatment? A study of self-generated treatment and recovery goals. Journal of Psychiatric Practice®, 25(2), 148–155. 30849065 10.1097/PRA.0000000000000369

[bibr34-00207640231189424] Nicholson J. Carpenter-Song E. A. MacPherson L. H. Tauscher J. S. Burns T. C. Lord S. E. (2017). Developing the WorkingWell mobile app to promote job tenure for individuals with serious mental illnesses. Psychiatric Rehabilitation Journal, 40(3), 276. 27322395 10.1037/prj0000201 PMC7480984

[bibr35-00207640231189424] Peters M. D. Marnie C. Tricco A. C. Pollock D. Munn Z. Alexander L. McInerney P. Godfrey C. M. Khalil H. (2020). Updated methodological guidance for the conduct of scoping reviews. JBI Evidence Synthesis, 18(10), 2119–2126. 33038124 10.11124/JBIES-20-00167

[bibr36-00207640231189424] Sansone R. A. Sansone L. A. (2012). Employment in borderline personality disorder. Innovations in Clinical Neuroscience, 9(9), 25. PMC3472897 23074700

[bibr37-00207640231189424] Shepherd G. Lockett H. Bacon J. Grove B. (2012). Establishing IPS in clinical teams: Some key themes from a national implementation programme. Journal of Rehabilitation, 78(1), 30–36.

[bibr38-00207640231189424] Soloff P. H. (2021). Bridging the gap between remission and recovery in BPD: Qualitative versus quantitative perspectives. Journal of Personality Disorders, 35(1), 21–40. 10.1521/pedi_2019_33_419 PMC9237745 30785863

[bibr39-00207640231189424] Thompson R. J. Payne S. C. Horner M. T. Morey L. C. (2012). Why borderline personality features adversely affect job performance: The role of task strategies. Personality and Individual Differences, 52(1), 32–36.

[bibr40-00207640231189424] Torrey W. C. Mueser K. T. McHugo G. H. Drake R. E. (2000). Self-esteem as an outcome measure in studies of vocational rehabilitation for adults with severe mental illness. Psychiatric Services, 51(2), 229–233. 10655008 10.1176/appi.ps.51.2.229

[bibr41-00207640231189424] Trevillion K. Stuart R. Ocloo J. Broeckelmann E. Jeffreys S. Jeynes T. Allen D. Russell J. Billings J. Crawford M. J. Dale O. Haigh R. Moran P. McNicholas S. Nicholls V. Foye U. Simpson A. Lloyd-Evans B. Johnson S. Oram S. (2022). Service user perspectives of community mental health services for people with complex emotional needs: A co-produced qualitative interview study. BMC Psychiatry, 22(1), 1–8. 10.1186/s12888-021-03605-4 35081929 PMC8791764

[bibr42-00207640231189424] Weinberg I. Ronningstam E. Goldblatt M. J. Schechter M. Maltsberger J. T. (2011). Common factors in empirically supported treatments of borderline personality disorder. Current Psychiatry Reports, 13, 60–68. 21057901 10.1007/s11920-010-0167-x

[bibr43-00207640231189424] Winsper C. (2021). Borderline personality disorder: Course and outcomes across the lifespan. Current Opinion in Psychology, 37, 94–97. 33091693 10.1016/j.copsyc.2020.09.010

[bibr44-00207640231189424] Zanarini M. C. Frankenburg F. R. Reich D. B. Fitzmaurice G. (2010). The 10-year course of psychosocial functioning among patients with borderline personality disorder and axis II comparison subjects. Acta Psychiatrica Scandinavica, 122(2), 103–109. 20199493 10.1111/j.1600-0447.2010.01543.x PMC3876887

