# Supplementary material for: Employment interventions to assist people who experience borderline personality disorder: A scoping review
Source: Int J Soc Psychiatry. 2023 Jul 27;69(8):1845–55. doi: 10.1177/00207640231189424 (PMC10685697; doi:10.1177/00207640231189424)

Supplementary File: Search Strategy for Medline

| Medline  14/4/22 = 83  Limit to English language and humans | Borderline personality disorder/OR  (((Borderline or impulsive personality) adj 1 type*) OR ((Borderline personality OR emotionally unstable personality OR borderline) adj1 disorder*) OR Cluster B). ti,ab,kf  AND  Employment/ OR  Rehabilitation, Vocational/OR  ((individual placement and support) or employment or vocation*). ti,ab,kf |
| --- | --- |


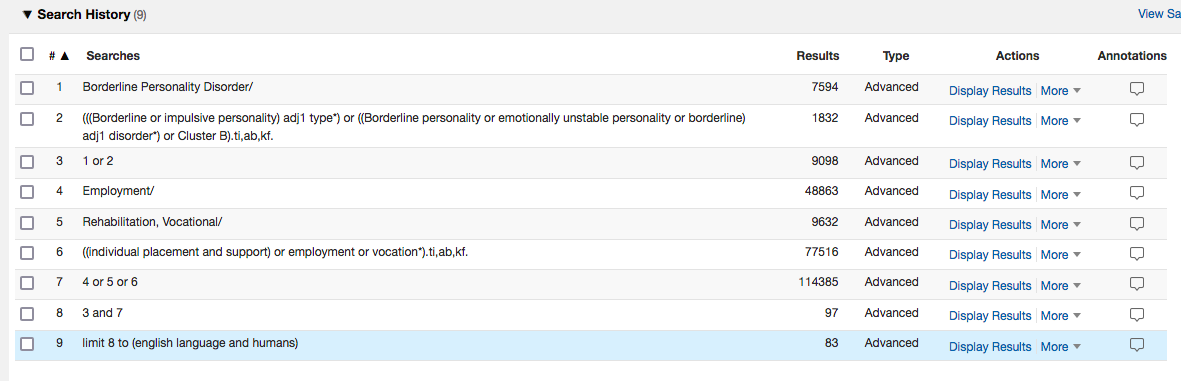

Supplement: sj-docx-1-isp-10.1177_00207640231189424 – Supplemental material for Employment interventions to assist people who experience borderline personality disorder: A scoping review [file sj-docx-1-isp-10.1177_00207640231189424.docx]
